# How clinicians make decisions about CTOs in ACT: a qualitative study

**DOI:** 10.1186/s13033-018-0230-2

**Published:** 2018-09-22

**Authors:** Hanne Kilen Stuen, Anne Landheim, Jorun Rugkåsa, Rolf Wynn

**Affiliations:** 1 Norwegian National Advisory Unit on Concurrent Substance Abuse and Mental Health Disorders, Innlandet Hospital Trust, Brummundal, Norway; 2 Department of Clinical Medicine, Faculty of Health Sciences, UiT The Arctic University of Norway, Tromsø, Norway; 3 Norwegian Centre for Addiction Research, University of Oslo, Oslo, Norway; 4 Health Services Research Unit, Akershus University Hospital, Lørenskog, Norway; 5 Centre for Care Research, University of South-Eastern Norway, Porsgrunn, Norway; 6 Divison of Mental Health and Addictions, University Hospital of North Norway, Tromsø, Norway

**Keywords:** Assertive community treatment, Coercion, Community treatment orders, Psychosis, Compulsory medication

## Abstract

**Background:**

The first 12 Norwegian assertive community treatment (ACT) teams were piloted from 2009 to 2011. Of the 338 patients included during the teams’ first year of operation, 38% were subject to community treatment orders (CTOs). In Norway as in many other Western countries, the use of CTOs is relatively high despite lack of robust evidence for their effectiveness. The purpose of the present study was to explore how responsible clinicians reason and make decisions about the continued use of CTOs, recall to hospital and the discontinuation of CTOs within an ACT setting.

**Methods:**

Semi-structured interviews with eight responsible clinicians combined with patient case files and observations of treatment planning meetings. The data were analysed using a modified grounded theory approach.

**Results:**

The participants emphasized that being part of a multidisciplinary team with shared caseload responsibility that provides intensive services over long periods of time allowed for more nuanced assessments and more flexible treatment solutions on CTOs. The treatment criterion was typically used to justify the need for CTO. There was substantial variation in the responsible clinicians’ legal interpretation of dangerousness, and some clinicians applied the dangerousness criterion more than others.

**Conclusions:**

According to the clinicians, many patients subject to CTOs were referred from hospitals and high security facilities, and decisions regarding the continuation of CTOs typically involved multiple and interacting risk factors. While patients’ need for treatment was most often applied to justify the need for CTOs, in some cases the use of CTOs was described as a tool to contain dangerousness and prevent harm.

## Background

In Norway, as in many other Western countries, there has been a substantial reduction in the number of inpatient beds and a move towards community-based services, referred to as a ‘deinstitutionalization’ of psychiatric care [1, 2]. As part of this process of deinstitutionalization, the primary locus of treatment of severe mental illness (SMI) shifted from hospitals to the community. Different legal mechanisms, such as community treatment orders (CTOs), have been used to compel treatment adherence in more than 75 jurisdictions worldwide [3]. Besides mandating patients with SMI to adhere to treatment, the CTO regime also allows for a rapid recall to hospital for its enforcement. In Norway, CTOs have been widely used since the implementation of the Mental Health Care Act (MHA) in 1961 [4]. Despite lack of robust evidence of effectiveness [5], Norwegian figures suggest that more than a third of all patients discharged from involuntary admissions in 2014 were placed on CTOs [6]. Although two national action plans to reduce the use of coercion have been launched, the involuntary admission and CTO rates in Norway have remained relatively high (61/100,000) compared to other Western countries since recording of data started in 2007 [7].

Decisions regarding compulsory interventions in general adult psychiatry are made by psychiatrists and authorized clinical psychologists [Responsible Clinicians (RCs)]. The legal criteria for involuntary hospitalization and CTOs, which are identical, are based on the presence of severe mental illness (SMI) [8]. CTOs have been referred to as a less restrictive treatment option than involuntary hospitalization, either to improve or restore patients’ health or prevent significant deterioration in the very near future (‘treatment criterion’) [9]. Commitment may also be used if the person is likely to pose an obvious and serious risk to his/her own life or health or that of others (‘dangerousness criterion’). Additionally, voluntary treatment must have been attempted (or obviously futile), and unless the ‘dangerousness’ criterion is met, compulsory mental health care must clearly appear to be the best option for the patient. RCs are required to conduct assessments every 3 months to consider whether the legal criteria are met. Decisions regarding involuntary hospitalization and CTOs do not include medication, and the responsible psychiatrist can initiate a separate medication order. Commitment decisions may be appealed to an independent Supervisory Commission, which conducts documentary controls every third month. If extended commitment after the initial 12 months is requested, the Supervisory Commission makes an independent review.

There is an ongoing discussion as to whether the mandatory element of the CTO produces greater clinical benefits for patients than offering them the same package of services on a voluntary basis [5, 10, 11]. International and Norwegian studies show that the use of CTOs is often justified on the basis of patients’ lack of illness insight [12], and that they are mainly used to provide support and treatment following involuntary hospital admissions [12–14]. Studies show that patients have mixed views of coercion in general and CTOs in particular, while psychiatrists and patients’ relatives are more positive [15–17]. Stensrud et al. [18] found that RCs in Norwegian services were worried about relapse, and therefore reluctant to make dynamic adjustments, even when patients were stable over time. Another Norwegian study [19] shows great variance in how RCs consider clinical and functional improvement. The duration of CTOs seems to depend on the RCs’ attitudes and opinions [13, 14, 19]. Although most clinicians would probably support a shift towards a more person-centred and collaborative decision-making approach, some studies have shown that many clinicians tend to use a more paternalistic type of argumentation in clinical decision-making situations regarding patients with SMI [18, 20, 21].

In a report from 2008, it was estimated that approximately 4000 people with SMI were not receiving appropriate mental health care [22]. In 2009 the Norwegian health authorities decided to fund the piloting of assertive community treatment (ACT) teams to provide services to this target group. The ACT model involves multidisciplinary teams with shared caseload responsibility and a low client–staff ratio (1:10), that provided flexible and intensive home-based support. Prior to this pilot, Norway had limited specialist ambulant community treatment outreach services.

Although ACT has been criticized for being paternalistic and coercive [23], studies show that patients generally are more satisfied with ACT than standard community mental health services [24, 25]. ACT targets patients with SMI with co-occurring substance abuse and poor social functioning, who are often difficult to engage in more traditional services. Some RCTs show that ACT improves outcomes, including reduced hospitalization, improved housing stability and treatment retention [26].

The research-based evaluation of the 12 first Norwegian ACT teams assessed clinical outcomes from 142 patients at baseline and after 2 years follow-up. Although there was no change in the overall number of admissions, there was a 50% reduction in both involuntary admissions and bed-days [27]. Among the 142 included patients, 32% were subject to CTOs at intake during the teams’ first year of operation.

The ACT model has a strong focus on promoting patients’ autonomy and recovery [28, 29], and, as we have shown previously, the enactment of CTOs typically involves competing priorities and role tensions [30]. Some studies show that assertive outreach teams reported using less intrusive approaches than other community mental health services [31, 32]. Studies have found wide variation in the use of CTOs [14, 33, 34], and we need more knowledge about responsible clinicians’ judgments and CTO follow-up decisions. The aim of this study was to explore how RCs within an ACT setting reason and make decisions about the continued use of CTOs, recall to hospital and the discontinuation of CTOs.

## Methods

### Design

Because CTO decisions and clinicians’ reasoning is an understudied area, we found a qualitative design, using a modified grounded theory approach, informed by a constructivist and interpretative framework to be appropriate [35]. Data consisted of in-depth interviews with RCs, case file reviews and observations of treatment planning meetings.

### Setting, sampling and recruitment

This study is part of the national evaluation of the first 12 Norwegian ACT teams [27]. When the present study started, the ACT teams had been established for 30 months, and they had showed moderate to high fidelity to the ACT model [29]. However, the ACT teams’ CTO rates varied, and after 30 months of operation, 6–52% of the patients were subject to CTOs. Participants were recruited purposively from four ACT teams that varied in size, in their use of CTOs and in how the CTO responsibility was organized, to ensure that we could include a wide range of experiences [35]. While the psychiatrists or clinical psychologists of the ACT teams in general were responsible for decisions about continued use of CTOs, recall to hospital and discontinuation of CTO, a few teams had chosen to leave the administrative CTO responsibility to clinicians at the Community Mental Health Centre (CMHC). When we started to recruit participants, 40 of 76 patients enrolled in Team 1 were on a CTO, and two full-time ACT psychiatrists were responsible for CTO decisions. Because one psychiatrist was responsible for most cases, this psychiatrist was interviewed twice. In Team 2, four of 67 patients were on a CTO, and the team psychiatrist and also a psychologist (on leave) were responsible for CTO decisions. In this team, there was a replacement of psychiatrists during the study period, and we decided to interview both psychiatrists. In Team 3, 23 of 68 patients were on a CTO, the ACT psychiatrist followed up patients with a compulsory medication order, while psychologists at the CMCH were responsible for decisions regarding the continued use of CTOs, recall to hospital and the discontinuation of CTOs.

In Team 4, 13 of 38 patients were under a CTO, and here we interviewed two psychologists from the CMHC, and also the team psychiatrist, who had prior experience with CTO decision making from other treatment settings. In a prior study involving patients from the same four teams [16], patients gave written consent to use their case notes in the present study. Team leaders and RCs who had been involved in administrative decision-making for the 15 participants in the patient study were given written information about the study [16]. All except three RCs took part. One was on maternity leave and two had only been involved for short periods in temporary positions, and were therefore not included. The final sample included eight RCs (two CMHC psychologists and six ACT psychiatrists); these were interviewed individually.

### Data collection

As a background for the individual interviews, we conducted case reviews of the files of 15 patients. After the patient interviews were conducted in 2013 [16], we followed the patients’ files until May 2015. In the individual interviews, participants were all asked to discuss specific CTO decisions, including the continued use of CTOs, recall to hospital and the discontinuation of CTOs. The interviews were based on a thematic interview guide developed by the authors, drawing on an assessment of relevant literature, previous patient interviews and associated case files [16]. Although the RCs were asked to describe and provide details of the 15 cases, many RCs also described other cases anonymously to provide typical examples of the content of the CTO, their reasoning and the clinical implications. We started all interviews with open questions about the enactment of CTOs and the organization of the follow-up responsibility. We also asked the RCs about the strategies applied to develop supportive relationships with the patients and about their perceptions of the CTO decision-making responsibility within an ACT context. The interviews were conducted at the CMHC and the ACT teams’ facilities. The individual interviews lasted 55–110 min. Approximately 1 year after the interviews, the first author (HKS) reviewed the 15 case files, before attending four theoretically sampled treatment planning meetings, one in each team. These were selected to observe team discussions and capture the clinical implications of different CTO decisions and how the CTO responsibility was organized [35]. The observations and the interviews were audio recorded and transcribed verbatim.

### Analysis of data

We used an iterative process of data collection to develop a conceptual understanding of the RCs’ reasoning and decision making based on categories grounded in the data [35]. In order to identify meaning units in the transcribed text, the two first interviews (from two different teams) were thematically coded. After reflections and thematic codes were written down, we made small adjustments to the interview guide to elicit richer data about the functional split between in- and outpatient care. Subsequently, we used the same approach for the other interviews. After the first initial coding, all the memos and the interviews were read in detail, to compare the most frequently used codes and to develop more focused codes. The most central codes were collapsed into broader categories by connecting sub-categories through the constant comparison of data, codes, categories and memos, to develop the range of properties and their dimensions (focused coding). Thereafter, we conducted an incident-by-incident coding of the treatment planning meetings, to clarify and extend the analytical categories. Subsequently, the categories were integrated and linked together (theoretical coding). Focused coding was performed manually, and subsequently NVivo software [36] was used to improve our overview of the data. The process of using a constant comparative method within and between categories was continued using the software until no new observations or properties emerged. Memos were written throughout the process, in order to increase the abstraction level and enhance the development of categories. The case files were used as a backdrop during the data collection. Although treatment planning meetings were important to consider, compare and specify team differences and the RCs’ reasoning, it is the interviews that are in the forefront in “Results” section.

## Results

In our analysis, we identified three main categories that reflected the overall finding ‘feeling more confident and secure through shared responsibility’: (1) CTO as a tool for achieving patient stability and safety, (2) CTO as a tool for containing dangerousness and preventing harm, and (3) CTO and ACT allowing for more nuanced judgments and reduced coercion. We discuss each in turn.

### CTO as a tool for achieving patient stability and safety

The participants described CTOs as a useful tool to ensure that patients remained in treatment. For patients with delusions or who for other reasons were not capable of making treatment decisions, the opportunity for prolonged use of a legal mechanism was seen as helpful.

*“I will continue the CTO as long as I possibly can. So, a certain time under a CTO, then one can try [voluntary treatment]. As long as I consider her to be so delusional, I won’t remove the CTO. That will be up to the Supervisory Commission to decide.”*

Many patients had an extensive history of treatment discontinuation and frequent readmissions. The RCs often wanted patients to remain on medication for 1–2 years to become stabilized. Few guidelines for decision-making existed, so participants relied on their clinical judgment of patients’ present and past situation to assess the best way to proceed.

“*I usually look at the past year and the so*-*called deterioration criterion. How likely is it that they’ll get so much worse that they can’t cope if we remove the CTO? We consider whether they understand that they need medication. So it’s a matter of going through what’s happened this past year, if things have been stable.*”

Although the team could not compel patients to take medication unless a valid medication order was in place, the RCs described how they tried to persuade, negotiate or make agreements. For instance, one RC described how she had spent several years on trials and errors with medications with a patient including deferring the medication administration to the hospital for a period after several violent incidents in the patient’s flat. Eventually, they succeeded in stabilizing the patient, and this was partly attributed to finding a more effective and tolerable medication regimen, the team’s long-term commitment, and a clear division of responsibilities.

*“From being in hospital 90% of the year, she’s had two short admissions during the last 2* *years. There’s been great collaboration around her [residential staff, hospital clinicians and ACT], where she’s been able to try things out. But it takes time to succeed. She may not have any more insight into her illness, but at least she’s more motivated for treatment.”*

Patients’ insight into their illness was presented as interdependent with the quality of the patient-clinician relationship, which in turn informed clinical judgments.

*“It seems unnecessary to continue the CTO in her [another patient’s] case from my point of view. With her I manage to collaborate about medication, and use of CTOs is then by definition not justified.”*

The decision to terminate the CTO was also founded on improved functioning and that the patient gradually had regained capacity to make informed treatment decisions.

*“She finds … that she has her own identity, a self, and she seems more capable of sorting out what she perceives as psychotic symptoms. I think she’s managed very well.”*

The CTO decision-making process must balance clinical needs and control of risk. The RCs emphasized that this made decision making complex, particularly when there was clinical uncertainty.

*“I’m often afraid that something will happen because I’ve reduced the medication. (X) is one example. He’s previously been sentenced for violence, he takes drugs and threatens all kinds of stuff. In his case, I’ve agreed to gradually reduce his medications. There’s no sign of active psychosis in his case file for the last years. He has strong side effects, refuses to take his medicine, and the police get involved. He is under a compulsory medication order, and it’s one hell of a mess. So now we’ve started to reduce his Cisordinol dose, and my plan is to continue to reduce his medication until we maybe see signs that he’s getting worse. This (medication withdrawal) doesn’t agree with the expectations of the specialist wards. He’s one example”*

### CTO as a tool for containing dangerousness and preventing harm

For patients with concurrent substance abuse, fluctuating illness severity and a history of violence, the participants agreed that CTOs combined with ACT could be justified as a long-term safety measure to prevent harm. They described the decision to use a CTO as founded on an overall consideration of the situation and a responsible prediction of future risk, involving deliberate self-harm, aggressive behavior or violence.

*“If it’s a matter of patients that have been severely ill, and committed serious violence and previously stopped taking their medicine as soon as the CTO was terminated, and they carry on taking [illicit] drugs, then I might keep the CTO for years if voluntary treatment doesn’t seem feasible.”*

There was substantial variation in how the RCs discussed the legal dangerousness criterion. In some cases the RCs described repeated patterns of neglect and risk to self or others. The participants’ accounts showed substantial overlap between their interpretation of the treatment criterion and the dangerousness criterion. While some RCs explained that they mainly used the dangerousness criterion when patients had to be readmitted to hospital, a few RCs used the dangerousness criterion to justify a CTO more than others. However, as the following example shows, most agreed that it should only be used when necessary.

*“I think it’s pretty ok to have him under coercion, because coercion protects society, but we see that it’s really difficult to build a therapeutic relationship with him. He’s actually a good example of the difficult considerations involved. Without the CTO, he’d just go to pieces. It would be quite reckless not to use a CTO with him.”*

However, according to some RCs, it was unclear where the legal threshold for obvious and serious danger was.

*“The dangerousness criterion is difficult to apply. With (X) it was a borderline case. You often solve it by writing something about it in the documents about the decision. He had a weapon and we considered there was a certain risk he might use it. You’re often asked [by the Supervisory Commission] if you want to apply it [the dangerousness criterion], but as I understand it, there’s a high threshold for dangerousness, so you often apply other criteria instead.”*

For clinicians with authority to make decisions about compulsion who were not regular members of the treatment team, the lack of knowledge of the individual patient was a significant problem in the prediction of risk.

*“As for assessing the risk of violence, it’s incredibly difficult to say how big the risk is that something will happen. That’s very hard to predict. (…) It might be easier if you were in a treatment position, within a team. But I’m sitting in my office, and I’m supposed to make an assessment of a patient I hardly know.”*

The participants emphasized the importance of the context in which psychiatric evaluations were made. Even if collaboration with the local treatment facilities and psychiatric hospitals had been established, many RCs considered the functional split between in- and outpatient care as a challenge. Concerns were that the responsible ward clinician might initiate treatment that had previously failed or that the ACT psychiatrist was not involved in CTO discussions prior to discharge. As one ACT psychiatrist said: “*We can make suggestions, but since they’re legally responsible [while patients are admitted], they make all the decisions*”. A further concern was that many patients with concurrent substance abuse were prematurely discharged from the ward by the responsible inpatient clinician. In one such case with a man in his early twenties, who was often not at home and difficult to reach, the RC strove to balance care and control.

“*I made a transfer because I didn’t want to have sole responsibility as long as he has a serious mental illness and makes these choices [substance abuse/crime]. When he was an inpatient, they didn’t find anything, so he was discharged on the same principles. He is not at home, has no phone and we run after him, knock on his window, and then we’re responsible for him. I think that’s difficult. I don’t like being responsible for someone I can’t get hold of at all.”*

One team psychiatrist explained that patients with co-occurring substance use disorders were referred to a separate dual diagnosis team, and that she often managed to arrange need-based long-term hospital admissions. Other RCs described a different scenario; lack of beds and inter-agency collaboration for the most severely ill patients was seen as a major challenge.

*“Our whole group, or our main group, which has suicidality, violence and substance abuse, has no sub*-*acute services. We don’t often get acute admissions for more than 2 or 3* *days.”*

Some RCs referred to a small subgroup of patients who had frequent encounters with the police, who were regularly transported to the acute ward, where the lack of psychiatric beds and inter-agency collaboration and increased professional liability put the clinicians’ professional responsibility on test. As one RC noted: “*You’re expected to keep the situation under control, which implies that you maybe ought to have people on CTOs. For me, that’s difficult”.*

### Use of CTOs and ACT allows for more nuanced judgments and reduced coercion

The RCs underlined that the team approach and the focus of the ACT model on assertive engagement strategies and comprehensive service provision allowed for more nuanced judgments and increased flexibility when working with patients with chaotic lives.

“*As a team we have better opportunities to adequately address the patients’ needs and provide close follow*-*up. I also find that we have more material to help us decide whether or not a patient should be on a CTO, and also that it’s easier to terminate the CTO. (..) Because many of us know the patients, our discussions become more composed, and more nuanced.*”

Frequent patient contact and the opportunities for this contact to remain over considerable time were presented as important in reducing coercion. Feeling more confident and secure through shared responsibility was a typical way of summing this up:

“*In my view, the most important things to reduce coercion are close contact, continuous follow*-*up care and to have good relationships*”.

“*We feel more confident about terminating a CTO in an ACT team.”*

However, conflicting attitudes and disagreements in the team, often involving medications, were challenges the team had to manoeuver. One psychiatrist who had been in ACT since the team was established described a steep learning curve. Critical team reflections and debriefings were considered as important learning arenas.

“*People joined the team with strong objections to overmedication. I was really frustrated, and also discouraged and afraid. (..). When people have been around and got some experience of the sickest patients, that kind of ideological attitude disappears. When it comes to individual patients, I’d say we do that [discuss medication]. We have lively discussions about medication, we look critically at the dose, and think about when we should start and how long we should wait.*”

While daily team meetings provided an overview of each patient’s condition, in teams where the team psychiatrist or psychologist was responsible for CTO follow-up decisions, the team was also more actively involved in CTO discussions. As one RC said:

“*We try to have a discussion. We look at the medical history and sum up about the patient, and then we ask everyone if they have an opinion for or against. We assess it together*.”

Close follow-up care and frequent observations were used to devise a colour scheme on the teams’ blackboard with details of all the patients; different colors indicated each patient’s current situation, legal status and treatment needs. According to participants, the ACT approach afforded flexibility to provide more intensive treatment to patients in periods of extra need, including illness severity and stressful life events. It also allowed for discussions of priorities, such as whether two instead of one ACT provider should conduct home visits.

“*How frequently we go and see patients depends on whether they’re in a green, yellow or red phase [on the blackboard]. If they’re actively psychotic and need close monitoring, they’re red*”.

To give an example of the team’s discussion of priorities and tailored interventions, the RC described a newly enrolled patient who several ACT providers had visited regularly in a high-security ward. During the first weeks following discharge, the team had daily conversations with both the patient and the residential staff, and this was gradually reduced to 2 days a week. The team psychiatrist had recently been involved to consider further safety measures, and decided to bring the patient back to the high-security facility after serious threats against residential staff. After a few days the patient had called the team to ask for support and assistance at discharge. In this case the RC considered the team’s close follow-up and monitoring as an alternative to long-term inpatient care.

*“We referred him back to the security ward on Friday and picked him up on Monday. Now he could be discharged with close follow*-*up care by the team. Coercion is still being used, but at a lower level than if we hadn’t been there for him.”*

## Discussion

This study illuminates the RCs’ reasoning surrounding the use of CTOs within an ACT setting, including decisions about their continued use, their termination, and recall to hospital, at a time when ACT represented a relatively new approach. The participants emphasized that CTO decisions involved tensions and challenging professional judgments. Although the use of CTOs was mainly founded on patients’ clinical needs, CTOs were also presented as a tool to contain dangerousness. The participants stressed that being part of a multidisciplinary team with shared caseload, frequent patient contact and comprehensive service provision allowed for more nuanced judgments and increased flexibility.

CTOs were typically described as a useful tool to ensure that patients remain in treatment, mainly to help patients achieve stability and remain safe, which the participants said often takes a long time. Patients’ symptom severity, lack of illness insight, co-occurring substance abuse, frequent readmissions and a history of deliberate self-harm or violence, and the likely consequences of patients’ decisions were presented as decisive factors in CTO decisions.

Norwegian and international studies show that CTOs are often founded on patients’ lack of insight, to prevent psychotic relapse [12], which often has a disruptive effect on people’s quality of life and their capacity for independence. A Norwegian study showed that CTO practices vary [19] and that some RCs only met patients at yearly reviews, knew little about the content of local care services and the impact of CTOs on patients’ everyday lives. Some studies suggest that clinicians’ narrow understanding of ‘lack of insight’ and that a one-sided focus on compliance with medication may impede patients’ recovery process [18, 37, 38]. Our data show that the ACT providers perceived that the multidisciplinary nature of their work allowed for frequent patient contact, coordinated support, relationship building and more flexible treatment options. As a specific component of clinical care, the ACT model’s focus on everyday activities and improving patients’ lives was seen as pivotal to improve patients’ well-being and to gradually help patients work toward greater independence and self-sufficiency [30]. Further, the ACT team’s long-term treatment perspective and the ways in which the teams often managed to gradually involve patients in treatment decisions and joint crisis planning were considered as important in reducing the use of coercion [39].

ACT targets patients with co-occurring substance abuse and low social functioning who have not remained in care. While some studies describe the implementation of CTOs as a complement to involuntary inpatient care [19, 40, 41], the participants in our study presented the use of CTOs combined with ACT as the least restrictive solution. The Norwegian ACT evaluation did not show a reduction in the number of admissions, but there was a significant reduction in compulsory admissions and total inpatient days. Patients with co-occurring substance abuse had significantly fewer involuntary inpatient days, despite severe problematic substance use at 2 years follow-up [42]. Although the ACT model has been criticized for being paternalistic and coercive, studies show that patients are generally more engaged and satisfied with ACT than traditional community-based services [25]. The Norwegian ACT evaluation also revealed a high user satisfaction; patients subject to CTOs were more satisfied with ACT than voluntarily enrolled patients [43], and they also reported the highest degree of recovery [44].

The participants emphasized that many patients that were referred to them from hospital wards and high-security facilities were already subject to CTOs and that the CTO decision-making in the ACT team typically involved multiple interacting and complex risk factors. Similar to what is reported in other studies, our participants emphasized that a multidisciplinary team approach and close monitoring provided more comprehensive understanding of risk [45]. In addition, the team’s position enabled the early detection of warning signs, quick responses and the stepping up of the intensity of interventions during crises all of which allowed for more focused preventative efforts.

The use of CTOs was sometimes presented as a long-term safety measure to prevent harm that could result from patients’ symptom severity, co-occurring substance abuse, frequent readmissions and a history of victimization, deliberate self-harm or violence. However, there was substantial variation in interpretation of the dangerousness criterion, and the two RCs that were not part of the treatment team expressed more doubts about the accurate prediction of risk. This “risk as difficult to predict” finding has been reported in other studies. Feiring and Ugstad [20] found that many RCs were reluctant to assess whether a patient was at risk of harming others or societal consequences of untreated mental disorders. Assessing patients’ potential dangerousness is also challenging for GPs [9]. In Norway, the MHA has been founded on a strong treatment philosophy [46], and clinicians may find it less stigmatizing to refer to patients’ treatment needs than to dangerousness.

Our study participants emphasized the importance of the context in which psychiatric assessments were made. While ACT allowed for comprehensive assessments, reflective team discussions and multiple interventions, the functional split between inpatient and outpatient care was seen as a challenge. Although the ACT teams were mainly involved in admissions and discharge, disagreements between the ACT psychiatrist and the responsible ward clinician and uncoordinated changes to treatment plans were typical concerns. An ongoing debate in other European countries concerns which of the organizational models in mental health care, i.e. continuity of care or a functional split of responsibility between inpatient and outpatient care, is most effective [47–49]. Lindgren et al. [50] found that continuity across treatment settings was associated with better long-term outcomes. Despite lack of solid evidence of whether specialization or continuity of care is more effective (length of stay) [48], studies show that patients and clinicians prefer continuity across inpatient and outpatient settings [48, 49, 51, 52].

Many RCs described lack of inter-agency collaboration regarding a small subgroup of patients with co-occurring substance abuse with higher risk of victimization and violence during acute phases of illness as a main concern. Bed pressure and lack of treatment resources was seen to place clinicians in a position where they have to balance discretionary standards for civil commitment and ambiguous benchmarks for what constitute good practice and acceptable risks. Based on a recent amendment in the Mental Health Care Act, from September 2017, involuntary admissions and CTOs can only be used if the person lacks decision-making capacity, unless the person is deemed to constitute a risk to his/her own life or the safety of others. The amendment also involves a narrowing of the dangerousness criterion, which no longer includes the risk of harm to the person’s own health. Although capacity is a cornerstone in autonomous decision making, it is well known that psychiatric patients’ capacity to make informed decisions can fluctuate [53]. Many RCs in our study stated that they often applied the treatment criterion to justify the need for CTOs. The RCs will now be required to use a more structured approach to consider patients’ capacity to make treatment decisions, as of yet we have no knowledge of whether the recent amendment influences RCs’ decisions within an ACT setting.

## Limitations

The strengths of this study is that it draws on a rich dataset, and that we know that participants work in ACT teams with moderate to high fidelity to the model. One limitation is the small number of participants. Although the aim of qualitative studies is not to generalize the results, we cannot exclude the possibility that other RCs would have provided a more nuanced and deeper understanding of the RCs’ reasoning and CTO decision making. Since a majority of the participants were psychiatrists, the inclusion of other participants may have enabled a focus on possible variations between professionals in attitudes and decision-making practices. It would have been interesting to complement this research with experiences of CTO decision making in an ACT setting from the perspectives of family members and hospital clinicians.

## Conclusions

The RCs found that many of the patients enrolled in ACT are hard to reach and difficult to treat. The ACT model’s holistic and long-term treatment perspective was described as a more attuned way of working with patients with complex needs than traditional outpatient services. The participants emphasized that CTOs were mainly founded on patients’ clinical needs, and also that establishing stability and safety for patients enrolled in ACT is often a lengthy process. The main finding, ‘feeling more confident and secure through shared responsibility’ illustrates that the focus of the ACT model on frequent patient contact, shared caseload and comprehensive service provision was considered as a major improvement on traditional community services in that it could facilitate more nuanced assessments and reduce coercion. However, in some cases the RCs described CTOs combined with ACT as a long-term safety measure to prevent harm. Many RCs described a small subgroup of patients with co-occurring substance abuse who had frequent encounters with the police, where bed pressure and lack of inter-agency collaboration was presented as a main challenge.

## Abbreviations

ACT: assertive community treatment
CMHC: Community Mental Health Centre
CTO: community treatment order
MHA: Mental Health Care Act
RC: responsible clinician
SC: Supervisory Commission
SMI: serious mental illness
